# Validity of a Novel Algorithm to Compute Spatiotemporal Parameters Based on a Single IMU Placed on the Lumbar Region

**DOI:** 10.3390/s25185822

**Published:** 2025-09-18

**Authors:** Giuseppe Prisco, Giuseppe Cesarelli, Maria Romano, Marina Picillo, Carlo Ricciardi, Fabrizio Esposito, Paolo Barone, Mario Cesarelli, Leandro Donisi

**Affiliations:** 1 Department of Medicine and Health Sciences, University of Molise, 86100 Campobasso, Italy; g.prisco2@studenti.unimol.it; 2 Department of Engineering, University of Naples Parthenope, 80133 Naples, Italy; giuseppe.cesarelli@uniparthenope.it; 3 Department of Information Technology and Electrical Engineering, University of Naples Federico II, 80125 Naples, Italy; maria.romano@unina.it (M.R.); carlo.ricciardi@unina.it (C.R.); 4 Surgery and Dentistry Scuola Medica Salernitana, Department of Medicine, University of Salerno, 84081 Salerno, Italy; mpicillo@unisa.it (M.P.); pbarone@unisa.it (P.B.); 5 Department of Advanced Medical and Surgical Sciences, University of Campania Luigi Vanvitelli, 80138 Naples, Italy; fabrizio.esposito@unicampania.it; 6 Department of Engineering, University of Sannio, 82100 Benevento, Italy; mcesarelli@unisannio.it

**Keywords:** gait analysis, gait event detection, inertial measurement units, sensor placement, spatiotemporal parameters, wearable sensors

## Abstract

Background: A single lumbar-mounted inertial sensor offers a practical alternative to optoelectronic systems for gait analysis, simplifying measurements and improving usability in the clinical field. However, its validity can be influenced by sensor placement and signal choice. This study aimed to develop and validate a novel algorithm for estimating spatiotemporal parameters using anteroposterior linear acceleration and angular velocity around the sagittal axis using a single inertial measurement unit (IMU) placed on the lumbar region. The proposed algorithm was validated comparing the parameters computed by the algorithm with the ones computed using a commercial wearable system based on a two-foot-mounted IMU configuration. Thirty healthy subjects underwent a 2 min walk test, and five spatiotemporal parameters were computed using the two methodologies. Study results showed that cadence and gait cycle time exhibited very high agreement, with only a small, statistically significant bias in cadence negligible for practical purposes. In contrast, swing, stance, and double-support parameters showed disagreement due to the presence of systematic proportional errors. This work introduces a novel algorithm for gait event detection and spatiotemporal parameter estimation, addressing uncertainties related to sensor placement, metric models, processing techniques, and signal selection, while avoiding synchronization issues associated with using multiple sensors.

## 1. Introduction

Human movement analysis is a multidisciplinary field focused on collecting quantitative data related to the mechanics of the musculoskeletal system during motor tasks [1]. The significance of motion analysis extends across multiple disciplines, including rehabilitation [2], sports science [3], ergonomics [4,5,6,7], and clinical diagnosis [8]. Within the field of human movement analysis, gait analysis focuses on the collection of quantitative data related to lower limbs [9]. Among the extractable quantitative information, spatiotemporal parameters are particularly important as they provide valuable insights into gait patterns and postural control. Traditionally, the gold standard for gait analysis is based on optoelectronic systems, but recent advances in technology, particularly in miniaturization and wearable sensor accessibility, have promoted a paradigm shift in the monitoring of motion biomechanics [10]. Wearable sensors based on inertial measurement units (IMUs) are nowadays a widely accepted alternative to optoelectronic systems [11,12,13]. The integration of wearable inertial systems coupled with dedicated signal processing algorithms for gait event detection is increasingly used in several scenarios, revolutionizing human movement monitoring.

As widely reported in the scientific literature, several signal processing techniques were proposed to detect gait events, starting from information extracted from IMUs placed in different parts of the body. Pappas et al. [14] developed a real-time gait event detection system based on three force-sensitive resistors mounted on the shoe insoles and a gyroscope able to measure the angular velocity of the foot around the sagittal axis. Jasiewicz et al. [15] explored three methodologies for gait event detection, using linear accelerations along sagittal and anteroposterior axes acquired from foot-mounted IMUs, angular velocity around the sagittal axis acquired by means of foot-mounted IMUs, and angular velocity around the sagittal axis acquired using shank-mounted IMUs. Li et al. [16] investigated the feasibility of linear acceleration signals along sagittal and anteroposterior axes acquired using a single IMU mounted on the shank to estimate walking speed. Lau et al. [17] assessed the feasibility of identifying gait events by evaluating a combination of sensor placement configurations (thigh, shank, and foot) and three signal components (anteroposterior acceleration, sagittal acceleration, and anteroposterior angular velocity). Zijlstra et al. [18] studied the feasibility of spatiotemporal gait parameter detection, based on a model of the body’s center of mass trajectory during walking, using one accelerometer placed at the L5 vertebrae. Gonzalez et al. [19] proposed an algorithm for the real-time detection of gait events based on anteroposterior accelerations acquired from one inertial sensor placed on the lower trunk. Digo et al. [20] compared three IMU setups using trunk anteroposterior acceleration, shank mediolateral angular velocity, and ankle mediolateral angular velocity, respectively, for the spatiotemporal gait parameter estimation. Cimolin et al. [21] investigated the feasibility of detecting valid spatiotemporal parameters in level walking in normal-weight and obese adolescents from lower trunk anteroposterior linear acceleration. Panero et al. [22] examined two different algorithms based on different setups using trunk anteroposterior linear acceleration and foot mediolateral angular velocity to estimate gait spatiotemporal parameters. Finally, Buganè et al. [23] investigated the feasibility of an algorithm based on anteroposterior linear acceleration acquired from the lower trunk to detect spatiotemporal parameters during level walking.

Despite the use of wearable inertial sensors coupled with specific algorithms for gait event detection and kinematic parameter estimation growing significantly in the field of motion analysis, studies demonstrating their validity are still few and discordant, underlining the potential uncertainty in gait parameter estimation due to the different sensor placements and the inertial signals considered to estimate kinematic parameters. In particular, single-sensor approaches using lumbar-mounted IMUs offer clear practical advantages in wearability and ease of use. However, previous studies have typically been limited to gait event detection or the estimation of a reduced set of spatiotemporal parameters. Moreover, their performance has not been comprehensively validated against reference systems, raising questions about robustness and generalizability.

In light of these considerations, this study aimed to develop and validate a novel algorithm for gait event detection and spatiotemporal gait parameter estimation based on anteroposterior linear acceleration and angular velocity around the sagittal axis, using a single IMU placed on the lumbar region. The spatiotemporal parameters computed using the proposed algorithm were compared with the corresponding ones computed by a commercial wearable system employing a two-foot-mounted IMU configuration. To assess the agreement between the two measurement approaches, statistical analyses including paired tests, Passing–Bablok (PB) linear regression, and Bland–Altman (BA) analysis were performed.

## 2. Materials and Methods

### 2.1. Wearable Inertial System for Gait Analysis

**Figure 1:**
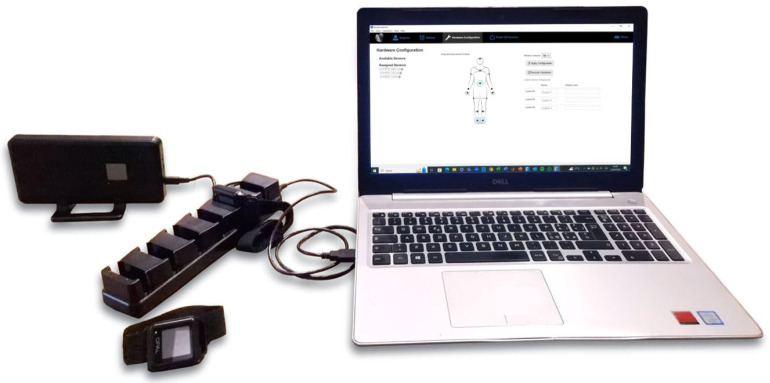
Mobility Lab system: access point, opal sensors, docking station, and Moveo Explorer.

The Mobility Lab system (APDM, USA) is a wearable inertial system that includes an access point, opal sensors, a docking station, and the Moveo Explorer software (Figure 1). The opal sensors, which are based on IMUs, integrate tri-axial accelerometers, gyroscopes, and magnetometers, recording linear acceleration and angular velocity signals at a sampling frequency of 128 Hz. The accelerometer has a 14-bit resolution, a bandwidth of 50 Hz, and a measurement range of ±200 g. The gyroscope features a 16-bit resolution, a bandwidth of 50 Hz, and a measurement range of ±2000°/s. The magnetometer has a 12-bit resolution, a bandwidth of 32.5 Hz, and a measurement range of ±8 Gauss. The dedicated software provides standardized protocols for quantitative movement analysis and also allows access to raw sensor signals. Previous studies have demonstrated the accuracy and repeatability of the system [24,25,26,27]. A single opal sensor placed on the lumbar region was used to compute spatiotemporal parameters using the proposed algorithm. The extracted parameters were then compared with those calculated by the Mobility Lab system using a two-foot-mounted configuration.

### 2.2. Study Population and Gait Protocol

Thirty healthy subjects (17 males and 13 females) with ages ranging from 21 to 60 years were enrolled. Their anthropometric characteristics are reported in Table 1. The present study was conducted in accordance with the guidelines of the Declaration of Helsinki and was authorized by the ethical committee of the coordinating center (ASL Napoli 3 Sud), n.0010177, 19 January 2021.

**Figure 2:**
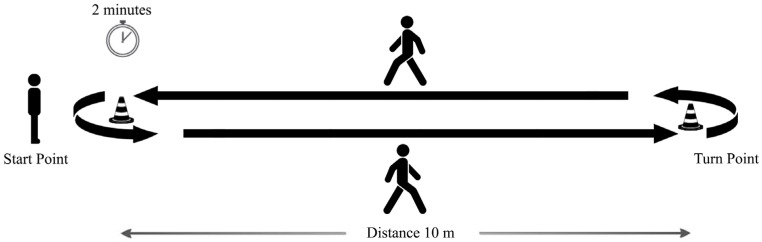
Experimental protocol: 2 min walk test.

**Figure 3:**
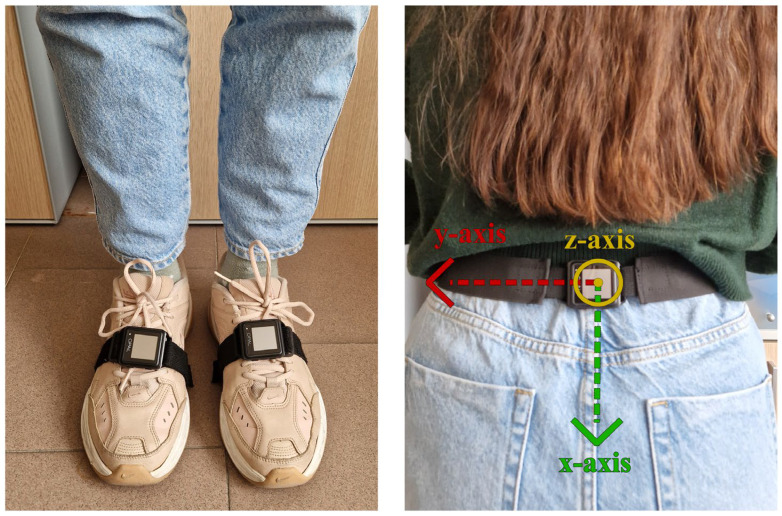
Sensor placement and local coordinate frames.

The subjects performed a standardized protocol, namely a “2 min walk test” included in the Moveo Explorer, which consists of a walk along a 10 m path forward and back for 2 min (Figure 2). Three IMUs were used: two placed on the feet, as required by the Mobility Lab system, and one placed on the lumbar region, according to the proposed algorithm. Data acquisition was performed by simultaneously using the two measurement systems for each participant, with a single trial recorded per subject. The experimental protocol, including task instructions and environmental setup, was standardized and identical across all participants. This design ensured that both measurement approaches were evaluated under the same conditions, minimizing potential biases related to measurement order or repeated trials. The reference coordinate frames of the lumbar IMU configuration are reported in Figure 3.

### 2.3. Data Processing and Gait Event Detection Algorithm

The proposed algorithms use the data acquired from a single opal sensor placed on the lumbar region. The anteroposterior (*z*-axis) acceleration and the angular velocity around the sagittal axis (*x*-axis) were used to detect gait events and estimate spatiotemporal gait parameters.

**Figure 4:**
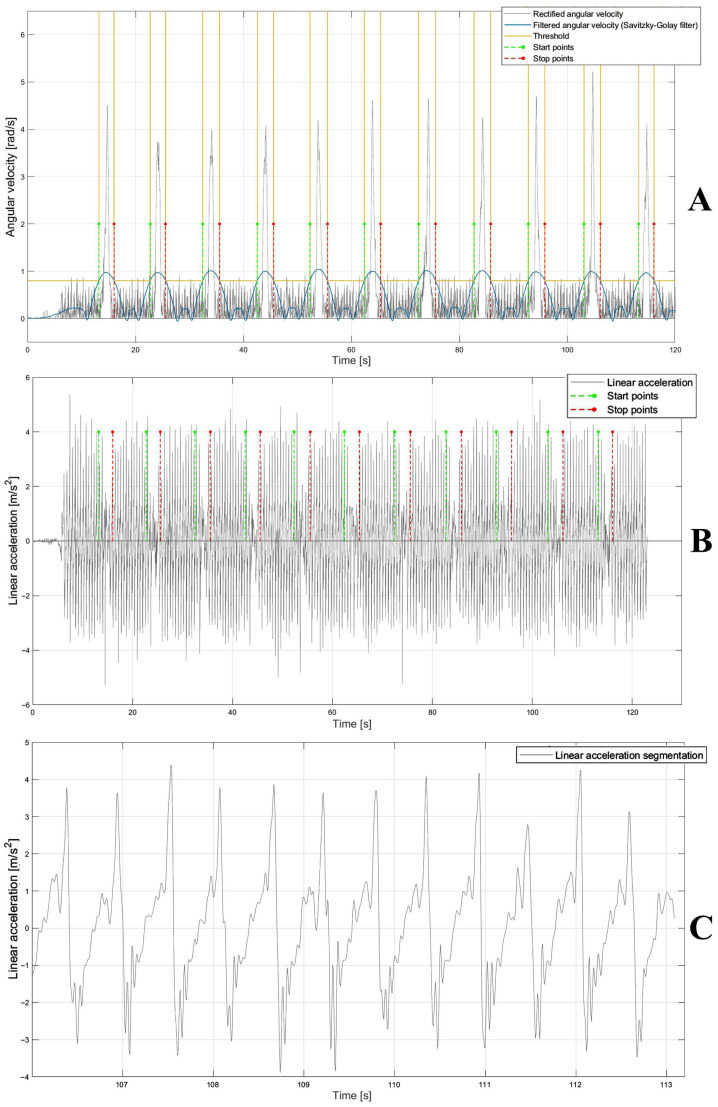
(**A**) Rectified angular velocity signal (gray), and rectified and filtered signal using Savitzky–Golay filter (blue) and threshold (yellow) to detect the start and stop points (green and red, respectively). (**B**) Linear acceleration (gray) and start and stop points (green and red, respectively). (**C**) Linear acceleration segmentation (gray) ranging from stop to start points (red and green, respectively) on which the temporal instants of heel-strike and toe-off were detected.

First, the detection of time intervals related to the turning phases was performed. To identify turns, the angular velocity signal was segmented to extract the time windows corresponding to the turning phases corresponding to an increase in the signal. The raw angular velocity data was first filtered through a 4th-order Butterworth band-pass filter (1–50 Hz) to remove the offset and preserve the spectral content of the inertial signal. The signal was then rectified and further filtered through a Savitzky–Golay filter [28] with a polynomial order equal to 2 and a frame length equal to 1101. Finally, an empirical threshold for each subject was established through an iterative approach to estimate the start and stop points in order to identify the time windows corresponding to the turning phases (Figure 4A). The time series of start and stop points were applied to the raw anteroposterior acceleration signal (Figure 4B) to identify the only time windows corresponding to 10 m walking phases, namely, the portions of the signal ranging from stop to start in which the gait events are detected (Figure 4C).

**Figure 5:**
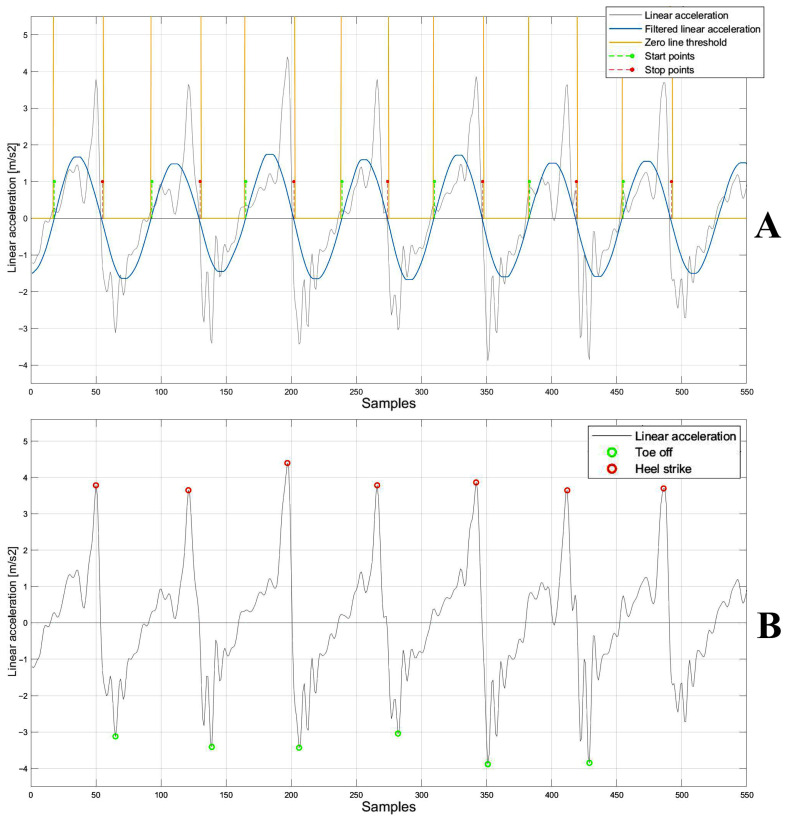
(**A**) Acceleration signal (gray), filtered signal using Savitzky–Golay filter (blue), zero-line threshold (yellow) and the start and stop points (green and red, respectively). (**B**) Linear acceleration signal (gray), heel-strike (red), and toe-off (green).

For each acceleration signal segment corresponding to 10 m (Figure 4C), the typical curve features represented by two positive peaks and one negative peak were detected to compute the time points corresponding to the gait events. The second positive peak and negative peak were identified as the heel-strike and toe-off events, respectively, [29]. For the temporal detection of peaks, the acceleration signal was first filtered using a 4th-order Butterworth band-pass filter (1–50 Hz). This was followed by the application of a Savitzky–Golay filter with a polynomial order equal to 50 and a frame length equal to 801. Notably, the optimal filter parameters were determined using a brute-force approach to identify the combination that most effectively segmented the acceleration signal within the Regions of Interest (ROIs). Finally, a 1D median filter with an order equal to 10 was used to complete the filtering process [30]. Filtering the signal allowed segmentation into negative and positive portions, facilitating the identification of individual gait events within the original signal (Figure 5A). Finally, by the intersection of the filtered signal with the zero-line threshold, new start and stop points—necessary to segment the acceleration signal in the ROI in which the temporal instants of gait events (heel-strike and toe-off) fall—were detected (Figure 5A). For each pair of ROIs, heel-strike (the second positive peak) was detected by identifying the maximum signal value within each ROI, ranging from start to stop, while the toe-off (the negative peak) was detected by identifying the minimum signal value within each ROI, ranging from stop to start (Figure 5B). Once the gait events were detected, the corresponding time points were extracted from the acceleration signal time series.

After identifying the times of the gait events, the following spatiotemporal parameters were calculated (formulas were written for the right leg; the same formulas were used for the left leg):

- Gait cycle time (GCT) [s] (1): the time between two consecutive heel-strikes of the same foot;

(1) $$GCT\left(k\right)={HS}_{r}\left(k+1\right)-{HS}_{r}\left(k\right)$$

- Cadence (C) [steps/min] (2): the number of strides in a minute;

(2) $$C=60\cdot \frac{s}{t}$$

- Stance phase (ST) [%] (3): the foot support phase, i.e., from heel-strike to toe-off of the same foot, with duration as a percentage of the gait cycle;

(3) $$ST\left(k\right)=\frac{{TO}_{r}(k)-{HS}_{r}(k)}{GCT(k)}\cdot 100$$

- Swing phase (SW) [%] (4): the foot swing phase, i.e., from toe-off to heel-strike of the same foot, with duration as a percentage of the gait cycle;

(4) $$SW\left(k\right)=\frac{{HS}_{r}\left(k+1\right)-{TO}_{r}(k)}{GCT(k)}\cdot 100$$

- Double-support phase (DS) [%] (6): the duration of the phase of support on both feet as a percentage of the gait cycle;

(5) $$IDS\left(k\right)=\frac{{TO}_{l}\left(k\right)-{HS}_{r}\left(k\right)}{GCT\left(k\right)}\cdot 100\;\;TDS\left(k\right)=\frac{{TO}_{r}(k)-{HS}_{l}(k)}{GCT(k)}\cdot 100$$

(6) $$DS\left(k\right)=IDS\left(k\right)+TDS(k)$$

where

- k: number of gait cycles;
- TO_r_: toe-off right;
- HS_r_: heel-strike right;
- s: steps;
- t: time of walk;
- IDS: initial double support;
- TDS: terminal double support;
- HS_l_: heel-strike left;
- TO_l_: toe-off left.

Finally, the spatiotemporal parameters computed for each subject were averaged for all the gait cycles and for both sides (left and right). While the formulas were originally designed for an IMU–shank configuration as reported in [31], in the current study, they were applied to an IMU–lumbar configuration.

### 2.4. Statistical Analysis

To verify the agreement between the two approaches, a two-tailed paired test, PB linear regression, and BA analysis were performed.

A parametric (*t*-test) or non-parametric (Wilcoxon test) paired test was implemented according to the Shapiro–Wilk normality test result [32].

PB linear regression allows assessing proportional and constant systematic errors. Systematic errors, both constant and proportional, are identified by means of the intercept and slope of the regression line. In particular, a statistically significant constant systematic error occurs when the 95% confidence interval of the intercept does not include the 0 value, while a proportional one occurs when the 95% confidence interval of the slope does not include 1 [33].

BA analysis evaluates the mean of the difference vector (bias) and the limits of agreement computed as bias ± 1.96 times the standard deviation (std) of the difference vector. The distribution of the data points in the BA plot helps to identify systematic errors (both constant and proportional). A random distribution around the 0 line implies agreement between the two methods, while the presence of trends is indicative of the presence of errors (i.e., systematic proportional errors if the trend is linear) [34]. Paired tests were carried out using JASP (JASP Team, version 0.17), while PB and BA analyses were performed using MATLAB (The MathWorks, version 2021b). For all tests, the confidence level was set at 95%.

## 3. Results

Table 2 shows the results of the paired test for each spatiotemporal parameter computed using the two methods, reported as mean ± std. The paired test (Table 2) showed that cadence was the only parameter with a statistically significant difference between the two methods (*p* < 0.05), with the proposed algorithm slightly overestimating values compared to the Mobility Lab system. For all the other parameters, no significant differences were observed, suggesting overall consistency between the two approaches.

Table 3 reports the PB linear regression analysis results for each spatiotemporal parameter, reporting the slope (m), the intercept (q), and their respective 95% confidence intervals. PB regression (Table 3) showed the absence of both systematic constant and proportional errors for cadence and gait cycle time, with slopes m close to 1 and intercepts q close to 0. In contrast, the stance, swing, and double-support phases showed 95% confidence intervals for the slopes m that did not include 1, indicating the presence of proportional systematic errors.

Table 4 shows the results of the BA analysis for each spatiotemporal parameter reporting the 95% confidence interval of the bias and the BA limits of agreement. BA analysis (Table 4) supported these findings: cadence showed a small but statistically significant positive bias, while gait cycle time displayed negligible bias with narrow limits of agreement.

**Figure 6:**
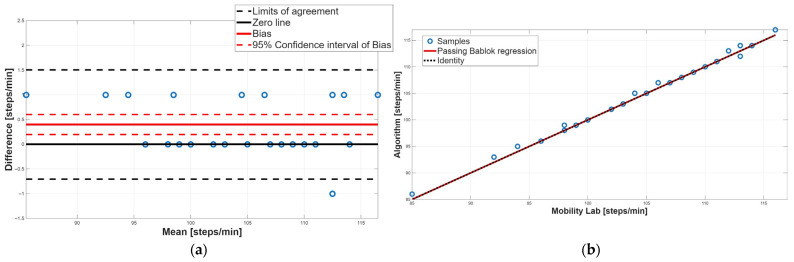
Cadence. (**a**) Bland–Altman plot with bias, zero-line, and limits of agreement. (**b**) Scatter plot with Passing–Bablok linear regression line and line of identity.

**Figure 7:**
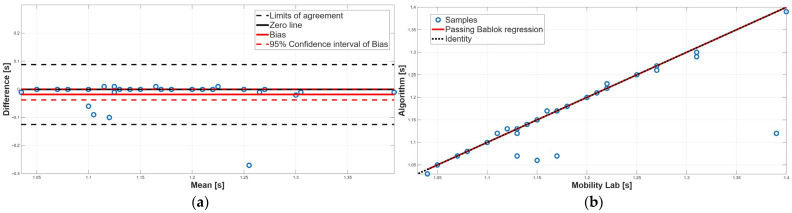
Gait cycle time. (**a**) Bland–Altman plot with bias, zero-line, and limits of agreement. (**b**) Scatter plot with Passing–Bablok linear regression line and line of identity.

**Figure 8:**
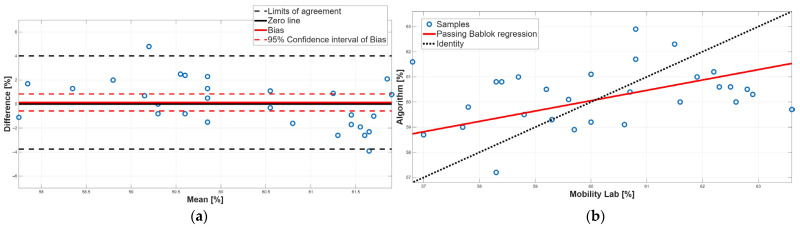
Stance phase. (**a**) Bland–Altman plot with bias, zero-line, and limits of agreement. (**b**) Scatter plot with Passing–Bablok linear regression line and line of identity.

**Figure 9:**
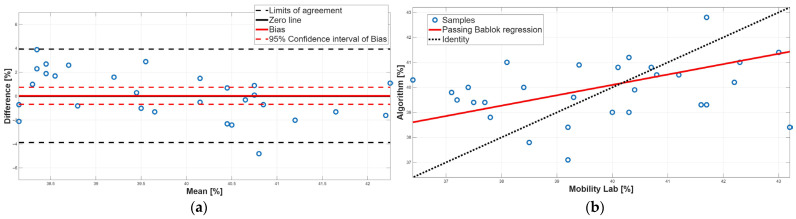
Swing phase. (**a**) Bland–Altman plot with bias, zero-line, and limits of agreement. (**b**) Scatter plot with Passing–Bablok linear regression line and line of identity.

**Figure 10:**
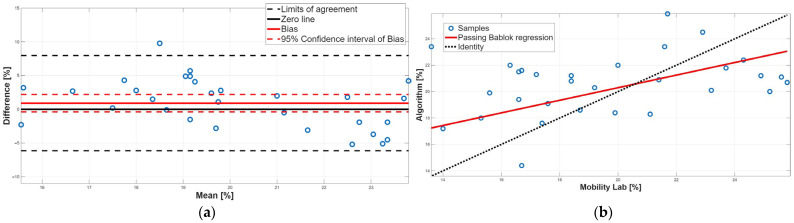
Double-support phase. (**a**) Bland–Altman plot with bias, zero-line, and limits of agreement. (**b**) Scatter plot with Passing–Bablok linear regression line and line of identity.

Figure 6, Figure 7, Figure 8, Figure 9 and Figure 10 show the PB and BA plots for all estimated spatiotemporal parameters. For stance, swing, and double-support phases, proportional systematic errors were evident, as reflected in the distribution trends in the plots (Figure 8, Figure 9 and Figure 10).

Concerning the cadence parameter, as evidenced by the paired test (Table 2), a statistically significant difference was found; in particular, the proposed algorithm overestimated the value of this parameter compared to the Mobility Lab system. The BA plot showed that most data points lie above the zero-line, confirming this overestimation; moreover, the 95% confidence interval of the bias did not include the 0 value, indicating the presence of a systematic constant error (Figure 6a, Table 4). Finally, the random distribution of the data points around the bias line excludes the presence of a proportional systematic error, as shown in Figure 6a. PB analysis confirmed the absence of a proportional systematic error, since the slope m of the linear regression line is perfectly equal to 1; conversely, it does not recognize the presence of a constant systematic error; indeed, the intercept q value was equal to 0 (Table 3). This contrasting result is due to the very low constant systematic error (0.3%). Overall, for the cadence parameter, an agreement between the two methodologies was assessed.

Regarding gait cycle time, no significant difference emerged from the paired test, suggesting an overall agreement (Table 2). PB analysis confirmed this result; indeed, the slope m and the intercept q of the linear regression line were equal to 1 and 0, respectively, confirming the absence of constant and proportional systematic errors (Table 3). Finally, BA analysis confirmed the absence of the two systematic errors, since the 95% confidence interval of the bias included the 0 value, and the distribution of the data points around the zero-line was random (Figure 7a, Table 4). Taken together, for the gait cycle time parameter, a perfect agreement between the two methodologies was found.

Concerning the stance phase, no statistically significant difference was found from the paired test, suggesting an overall agreement between the two methods (Table 2). This result was confirmed by the BA analysis; indeed, the 95% confidence interval of the bias contained the 0 value, assuring the absence of a constant systematic error (Table 4). However, BA analysis showed the presence of a proportional systematic error since a negative linear trend in the data point distribution was present (Figure 8a). The presence of this error was confirmed by the PB analysis; indeed, the slope m of the linear regression line was equal to 0.41, and its 95% confidence interval did not include the 1 value (Table 3). These findings indicate that, due to the presence of a proportional systematic error, no agreement between the two methods for the stance phase was found.

Similarly to the stance phase, for the swing phase, no statistically significant difference was found between the two methods (Table 2). BA analysis confirmed this result; indeed, the 95% confidence interval of the bias included the 0 value which implies the absence of a constant systematic error (Table 4). However, the BA analysis showed the presence of a proportional systematic error since the distribution of the data points in the BA plot presents a negative linear trend, as shown in Figure 9. This error was confirmed by the PB analysis; indeed, the 95% confidence interval of the slope m of the linear regression line did not include 1 (Table 3). Taken together, for the swing phase, there was disagreement between the two methods. This result was expected since the swing phase is complementary to the stance phase.

Finally, the double-support phase did not present a statistically significant bias, as shown in Table 2 and Table 4. However, there is a disagreement between the two methodologies because there is a proportional systematic error; indeed, as shown in Table 3, the 95% confidence interval of the PB linear regression slope m did not contain 1; this result was confirmed by the BA analysis, since in the BA plot the presence of a linear trend was found (Figure 10a). In conclusion, for the double-support phase, a disagreement between the two approaches was found due to the presence of a proportional systematic error. This result was to be expected since the double-support phase is correlated with the stance phase and the swing phase.

Table 5 summarizes the results of the agreement between the Mobility Lab system and the proposed algorithm for each spatiotemporal parameter.

## 4. Discussion

This study aimed to develop and validate an algorithm for estimating spatiotemporal gait parameters using anteroposterior linear acceleration and angular velocity around the sagittal axis acquired from a single IMU placed on the lumbar region. The validation analysis was carried out by comparing the spatiotemporal parameters computed using the developed algorithms and the ones calculated using the Mobility Lab system.

Study results showed the validity of the proposed algorithm for computing cadence and gait cycle time; similar results were not observed for the gait phase parameters, namely, the swing, stance, and double-support phases, for which proportional systematic errors were found. These parameters are well-established biomarkers of gait dysfunction. Indeed, prolonged double-support duration is recognized as an indicator of impaired stability and increased fall risk in Parkinson’s disease and in elderly people, whereas altered stance and swing durations are commonly used to assess gait asymmetry and dysfunction following stroke [35,36,37]. Although proportional errors may theoretically affect the estimation of gait phase parameters in pathological gait, the strong agreement observed for cadence and gait cycle time ensures that the algorithm retains substantial clinical utility, as these parameters alone provide robust indicators of gait health and functional mobility. Importantly, systematic proportional errors can be mitigated using a corrective regression model, which can be considered robust when at least 30 subjects are available, as in the case under study.

Several studies in the literature demonstrated poor agreement in estimating gait cycle phases using a single sensor placed at the lumbar level [21,23]. For instance, Cimolin et al. [21] studied the validity of spatiotemporal parameters computed using the G-Walk sensor (BTS Bioengineering, Garbagnate Milanese, Italy) placed on the lower trunk in obese adolescents, comparing it with an optoelectronic system (Vicon, Oxford Metrics, Yarnton, UK). The authors concluded that the inertial system was a reliable solution for evaluating spatiotemporal parameters, although BA analysis suggested a proportional systematic error for stance and double-support phases. Similarly, Bugané et al. [23] evaluated an inertial wireless system (Free4Act, LetSense Group, Castel Maggiore, Italy) based on a single sensor placed on the L5 vertebrae, comparing it with an optoelectronic Vicon system to identify gait spatiotemporal parameters during level walking. The authors concluded that calculating the parameters for step cycle phases was critical due to the difficulty in identifying the timing of gait cycle events, which was associated with the presence of double positive peaks. Both studies [21,23] utilized the anteroposterior component of the acceleration signal, with gait event times identified on the signal filtered using a low-pass Butterworth filter with a 2 Hz cutoff frequency. In contrast, this study applied filtering solely for the identification of ROIs, while the detection of gait event times (toe-off, heel-strike) was carried out on the original signal. This different approach ensures more precise detection of gait events, leading to more accurate spatiotemporal parameter estimation. Kvist et al. [38] explored the concurrent validity of three algorithms using lumbar and foot-mounted IMU data to calculate spatiotemporal gait parameters in a population of healthy adults: two methods utilized an inverted pendulum model, and the other one was based on strapdown integration. Consistent with our results, they concluded that for the inverted pendulum methods using the lumbar sensor data, cadence and step time showed excellent agreement, while double support exhibited poor agreement. Similarly, Aqueveque et al. [39] validated a portable system based on a single lumbar IMU combined with a mobile application. They reported reliable estimation of overall spatiotemporal parameters, particularly cadence and gait cycle time, but they noted that phase-specific measurements such as stance, swing, and double support were less precise. In contrast, Soulard et al. [40] demonstrated that foot-worn IMUs provide highly reliable measurements of gait parameters, even under dual-task conditions, due to their proximity to the primary sources of foot–ground contact events. These comparisons suggest that the limitations observed in stance, swing, and double support in our study are largely attributable to the single-lumbar-sensor configuration rather than the algorithm itself. Nevertheless, the strong agreement observed for cadence and gait cycle time confirms that our approach maintains clinical utility, offering a simplified and cost-effective solution for gait analysis that remains robust for evaluating global gait timing and functional mobility.

Other studies confirmed the poor agreement in extracting gait cycle phases, such as swing, stance, and double support, using the IMU–lumbar configuration, comparing two commercially available inertial wearable devices. For instance, De Ridder et al. [41] aimed to evaluate the validity of a commercially available body-worn sensor (G-Walk) for spatiotemporal gait parameters in a healthy population, using the GAITRite walkway system (CIR Systems, Franklin, NJ, USA) as the gold standard. The authors concluded that while excellent validity was demonstrated for cadence and gait cycle time, cautious interpretation was necessary for temporal parameters based on final foot contact (stance, swing, and double-support time). Moreover, Donisi et al. [24] studied the agreement between two commercial wearable inertial systems (Mobility Lab and G-Walk), considering several spatiotemporal parameters. The authors analyzed cadence, stride velocity, gait cycle time, stride length, stance, and swing phase, concluding that, except for gait cycle time and cadence, the two systems did not provide perfect agreement. Both studies [24,41] achieved similar results to the current one. In line with this work, the results achieved in this study indicated that the IMU–lumbar setup might be less effective in detecting gait cycle phases compared to configurations with sensors placed on the lower limbs. Parameters like gait cycle time and cadence, which describe overall body movement, are less influenced by sensor location than those related to gait phases (swing, stance, and double support). On the other hand, sensors positioned on the lower limbs, such as the feet and ankles, provide more detailed information about gait dynamics. Hence, the poor agreement about swing, stance, and double support phases cannot exclusively be attributable to the proposed algorithm but partly to the specific configuration chosen based on a single IMU placed on the lumbar region. However, several strategies may mitigate the proportional systematic errors observed in stance, swing, and double-support phases. Algorithmic adjustments can enhance gait event detection by employing adaptive thresholds that scale with walking speed or signal amplitude, and by combining multiple features derived from the lumbar sensor, including anteroposterior and vertical accelerations as well as angular velocity. Calibration-based approaches, including subject-specific calibration sessions or regression-based correction models, can further compensate for consistent proportional deviations. Additionally, post-processing methods informed by physiological constraints, such as ensuring that stance plus swing equals a complete gait cycle and that double-support durations remain within plausible ranges, can correct implausible values and reduce bias. Collectively, these strategies allow a single-lumbar-sensor setup to maintain simplicity, portability, and low cost while improving the accuracy of gait phase estimation. The effectiveness of these approaches should be confirmed through experimental validation under diverse walking conditions.

Although limited to spatiotemporal parameters, the proposed methodology can be performed in various environments thanks to the minimal configuration and short processing time. Using a single sensor significantly reduces both the cost and complexity of the analysis and eliminates the need for sensor synchronization, which is required in configurations such as the ankle setup that involve at least two sensors. This minimal setup not only enhances practicality for research and clinical applications but also enables telerehabilitation, remote monitoring, and sports performance assessment, where portable and easy-to-deploy systems are advantageous. The simplicity of the system and low cost further facilitate continuous gait monitoring in both clinical and non-clinical settings, providing timely feedback for therapeutic interventions or performance optimization. Moreover, this algorithm could be applicable for a range of clinical studies, such as identifying pathological gait disturbances or assessing the effects of therapeutic interventions. Moreover, this algorithm could be applicable for a range of clinical studies, such as identifying pathological gait disturbances or assessing the effects of therapeutic interventions.

The limitations of this study include a relatively small and homogenous sample based on 30 healthy and young participants, which may limit the generalizability of the proposed algorithms, especially for clinical applications. Indeed, in Parkinson’s disease, gait patterns may differ substantially, making it important to assess whether the algorithm can adapt to pathological signals. Additionally, another limitation can be represented by the simplified gait protocol involving only straight walking and rotations. To address these limitations, future studies will include larger and more heterogeneous cohorts, including patients with gait impairments, and incorporate more complex motor tasks to enhance statistical robustness, strengthen algorithm validation, and enable a more comprehensive evaluation of gait performance. Moreover, as future development, the proposed algorithm will be investigated for potential enhancements, with a particular focus on improving its ability to detect the phases of gait within the gait cycle through the use of all three acceleration components related to the lumbar region. Finally, future studies could focus on the impact of sensor misalignment on parameter estimation accuracy. Despite these planned improvements, the current results for gait parameter estimation are already very promising, indicating that the system can be effectively applied across various fields of human movement analysis.

## 5. Conclusions

The current study presented an algorithm to detect spatiotemporal gait parameters using a single IMU placed on the lumbar region and considering the anteroposterior acceleration to compute gait parameters and the angular velocity around the sagittal axis to eliminate turning phases during 2 min walking on a 10 m straight path. The algorithm was validated by comparing the spatiotemporal parameters computed using the proposed algorithm with those extracted by means of a commercial wearable inertial system for gait analysis (Mobility Lab). Although a disagreement was observed for gait phase parameters due to the presence of a systematic proportional error, probably due to the lumbar configuration as partially highlighted in the scientific literature, a very high agreement was found for global parameters such as cadence and gait cycle time. However, further validation is required by comparing the estimation of our algorithm with that obtained from a gold-standard gait analysis system based on optoelectronic technology. In addition, systematic proportional errors can be mitigated using appropriate corrective methodological approaches. In conclusion, the proposed algorithm may be considered a valid tool to integrate into motor performance assessment.

## Figures and Tables

**Table 1:** Anthropometric characteristics of the study population.

Anthropometric Characteristics	Mean ± Standard Deviation
Age (years)	35.50 ± 10.74
Height (cm)	171.50 ± 8.75
Weight (kg)	76.88 ± 17.88
Body Mass Index (kg/m^2^)	26.04 ± 4.41

**Table 2:** Paired test analysis.

Spatiotemporal Parameters	Algorithm (Mean ± Std)	Mobility Lab (Mean ± Std)	*p*-Value
Cadence [steps/min]	103.63 ± 7.44	103.23 ± 7.61	<0.05
Gait cycle time [s]	1.16 ± 0.09	1.18 ± 0.09	0.08
Stance phase [%]	20.58 ± 2.32	19.65 ± 3.60	0.23
Swing phase [%]	60.29 ± 1.16	60.16 ± 1.92	0.76
Double-support phase [%]	39.85 ± 1.17	39.81 ± 1.91	0.87

**Table 3:** Passing–Bablok linear regression analysis.

Spatiotemporal Parameters	m	95% CI_m	q	95% CI_q
Cadence [steps/min]	1.00	1.00 to 1.00	0.00	0.00 to 0.00
Gait cycle time [s]	1.00	0.97 to 1.00	0.00	0.00 to 0.03
Stance phase [%]	0.41	0.14 to 0.79	35.36	12.99 to 51.87
Swing phase [%]	0.41	0.17 to 0.83	23.79	6.96 to 33.23
Double-support phase [%]	0.48	0.20 to 0.86	10.76	4.38 to 16.47

Abbreviations: m: slope of the Passing–Bablok regression line; q: intercept of the Passing–Bablok regression line; 95% CI_m = slope 95% confidence interval; 95% CI_q = intercept 95% confidence interval.

**Table 4:** Bland–Altman analysis.

Spatiotemporal Parameters	Bias	95% CI_Bias	LOA
Cadence [steps/min]	0.40	0.20 to 0.60	−0.70 to 1.50
Gait cycle time [s]	−0.02	−0.04 to 0.00	−0.13 to 0.09
Stance phase [%]	0.13	−0.58 to 0.84	−3.75 to 4.01
Swing phase [%]	0.03	−0.68 to 0.75	−3.87 to 3.94
Double-support phase [%]	0.91	−0.38 to 2.20	−6.15 to 7.97

Abbreviations: 95% CI_Bias = bias 95% confidence interval; LOA = limit of agreement.

**Table 5:** Results of the agreement between the Mobility Lab system and the proposed algorithm for each spatiotemporal parameter.

Spatiotemporal Parameters	Level of Agreement	Type of Error
Cadence [steps/min]	Agreement	None
Gait cycle time [s]	Agreement	None
Stance phase [%]	No agreement	Proportional systematic error
Swing phase [%]	No agreement	Proportional systematic error
Double-support phase [%]	No agreement	Proportional systematic error

## Data Availability

The datasets generated and analyzed in this study are not publicly available due to the privacy policy but are available from the corresponding author upon reasonable request.

## Abbreviations

The following abbreviations are used in this manuscript:

IMU	Inertial measurement unit
ROI	Region of interest
GCT	Gait cycle time
C	Cadence
ST	Stance phase
SW	Swing phase
DS	Double support phase
k	Number of gait cycles
TO_r_	Toe-off right
HS_r_	Heel-strike right
s	Steps
t	Time of walk
IDS	Initial double support
TDS	Terminal double support
TO_l_	Toe-off left
HS_l_	Heel-strike left
PB	Passing–Bablok
BA	Bland–Altman
std	Standard deviation
m	slope
q	intercept
